# Supplementary material for: The Systems Biology Research Tool: evolvable open-source software
Source: BMC Syst Biol. 2008 Jun 29;2:55. doi: 10.1186/1752-0509-2-55 (PMC2446383; doi:10.1186/1752-0509-2-55)
Supplement: Additional file 1 — SBRT Archive. An archive of the current version of the Systems Biology Research Tool. [file 1752-0509-2-55-S1.zip › sbrt-1.4.0/doc/developers_guide/api/sbrt/ext/metatool/MetatoolCycleComputer.html]

MetatoolCycleComputer


---


|  |  |  |
| --- | --- | --- |
| **Overview** \| **Package** \| **Class** \| **Use** \| **Tree** \| **Deprecated** \| **Index** \| **Help** | |  |
| PREV CLASS   **NEXT CLASS** | **FRAMES**    **NO FRAMES**     **All Classes** |  |
| SUMMARY: NESTED \| FIELD \| CONSTR \| METHOD | DETAIL: FIELD \| CONSTR \| METHOD |  |


---


## sbrt.ext.metatool Class MetatoolCycleComputer

```
java.lang.Object
  sbrt.ext.metatool.MetatoolCycleComputer
```

**All Implemented Interfaces:**: CycleComputer

---

``` public class MetatoolCycleComputer extends java.lang.Object implements CycleComputer ```

This class is used to run `metatool` to compute steady state cycles.

**Author:**
:   This class was written and documented by Jeremiah Wright while in the
    Wagner lab.

---

**Constructor Summary**

`MetatoolCycleComputer(java.io.File executable)`
  
          Constructs a new metatool steady state cycle computer.


| **Method Summary** | |
| --- | --- |
| `SparseDoubleMatrix<?,java.lang.String>` | `getCycles(IrrevRxnFluxome fluxome)`             Returns the set of all steady state cycles contained in the provided fluxome. |
| `ProgressMonitor` | `getProgress()`             Returns a progress monitor for this cycle computer. |

**Methods inherited from class java.lang.Object**

`clone, equals, finalize, getClass, hashCode, notify, notifyAll, toString, wait, wait, wait`

**Constructor Detail**

### MetatoolCycleComputer

```
public MetatoolCycleComputer(java.io.File executable)
```

:   Constructs a new metatool steady state cycle computer.

    **Parameters:**: `executable` - the full name of the metatool executable.


**Method Detail**

### getProgress

```
public ProgressMonitor getProgress()
```

:   Returns a progress monitor for this cycle computer.

    :   **Specified by:**: `getProgress` in interface `CycleComputer`
    :   **Returns:**: a progress monitor for this cycle computer.

---


### getCycles

```
public SparseDoubleMatrix<?,java.lang.String> getCycles(IrrevRxnFluxome fluxome)
```

:   Returns the set of all steady state cycles contained in the provided
    fluxome.

    :   **Specified by:**: `getCycles` in interface `CycleComputer`
    :   **Parameters:**: `fluxome` - the fluxome for which all cycles will be returned. **Returns:**: the extreme current matrix. Column variables are reaction names, and each row represents a steady state cycle.


---


|  |  |  |
| --- | --- | --- |
| **Overview** \| **Package** \| **Class** \| **Use** \| **Tree** \| **Deprecated** \| **Index** \| **Help** | |  |
| PREV CLASS   **NEXT CLASS** | **FRAMES**    **NO FRAMES**     **All Classes** |  |
| SUMMARY: NESTED \| FIELD \| CONSTR \| METHOD | DETAIL: FIELD \| CONSTR \| METHOD |  |


---
